# Mature mediastinal teratoma with adhesions to the pericardium: A rare case report

**DOI:** 10.1002/ccr3.9552

**Published:** 2024-11-01

**Authors:** Somar Mansour, Ali Badr, Majd Mansour, Mouhamad Badr, Ali Afif, Samer Rajab, Zuheir Alshehabi

**Affiliations:** ^1^ Department of Pathology, Cancer Research Center Tishreen University Hospital Latakia Syria; ^2^ Faculty of Medicine, Cancer Research Center Tishreen University Latakia Syria; ^3^ Department of General and Thoracic Surgery Tishreen University Hospital Latakia Syria

**Keywords:** case report, mature, mediastinal, pericardial adhesion, teratomas

## Abstract

**Key Clinical Message:**

Mature mediastinal teratoma (MMT) is a benign tumor that is composed of well‐differentiated tissues from all three germ cell layers. Malignant tumors have a distinct feature of adhering to the surrounding organs. Therefore, adhesion of MMT to the adjacent tissues, as a benign tumor, is rare and considered a surgical challenge. It leads to incomplete tumor resection to avoid damage to the surrounding tissues.

**Abstract:**

MMT is a germ cell tumor that contains well‐differentiated tissues from ectodermal, mesodermal, and endodermal germ cell layers. MMT comprises approximately 3%–12% of mediastinal tumors in adults and 60%–70% of mediastinal germ cell tumors. Complete surgical resection is the standard treatment. When adhesion to the surrounding tissues is present, residual tissues could be retained to reduce the injury of the peripheral blood vessels and nerves. We are reporting a rare case of MMT with adhesions to the pericardium and discussing the diagnostic and surgical challenges.

## 1 INTRODUCTION

Mature teratomas are subtypes of germ cell tumors that contain tissues from endodermal, mesodermal, and ectodermal germ cell layers.^1^ These tumors typically arise from gonads. Mature teratomas in the mediastinum are rare lesions; they are typically detected in the anterior mediastinum which is the most common location of extragonadal germ cell tumors.^1^, ^2^ In addition, most teratoma tumors in the anterior mediastinum arise from the thymus or near thymus parenchyma, and intrapericardial or pericardial arising teratomas are considered extremely rare.^2^

Mediastinal teratomas most frequently occur in children and young adults. Patients are usually asymptomatic, but large tumors may present with symptoms of compression.^3^ Complete surgical resection is the standard treatment. When adhesion to the surrounding tissues is present, residual tissues could be retained to reduce the injury of the peripheral blood vessels and nerves.^4^

Herein, we report a rare case of mature mediastinal teratoma (MMT) with adhesions to the pericardium and discuss the diagnostic and surgical challenges.

## 2 CASE HISTORY

A 14‐year‐old girl was presented to the thoracic clinic by her parents with a complaint of chest pain radiating to the right shoulder. The pain started a month ago, was accompanied by intermittent dry cough and was not responding to acetaminophen. There were no systemic symptoms, hemoptysis or dyspnea. Family history revealed diabetes mellitus type II and hypertension. There was no medical or surgical history.

## 3 METHODS (DIFFERENTIAL DIAGNOSIS, INVESTIGATIONS, AND TREATMENT)

Physical examination showed diminished respiratory sounds on the right side of the chest. A chest X‐ray was performed and showed an opacity occupying the middle zone of the right hemithorax.

**FIGURE 1:**
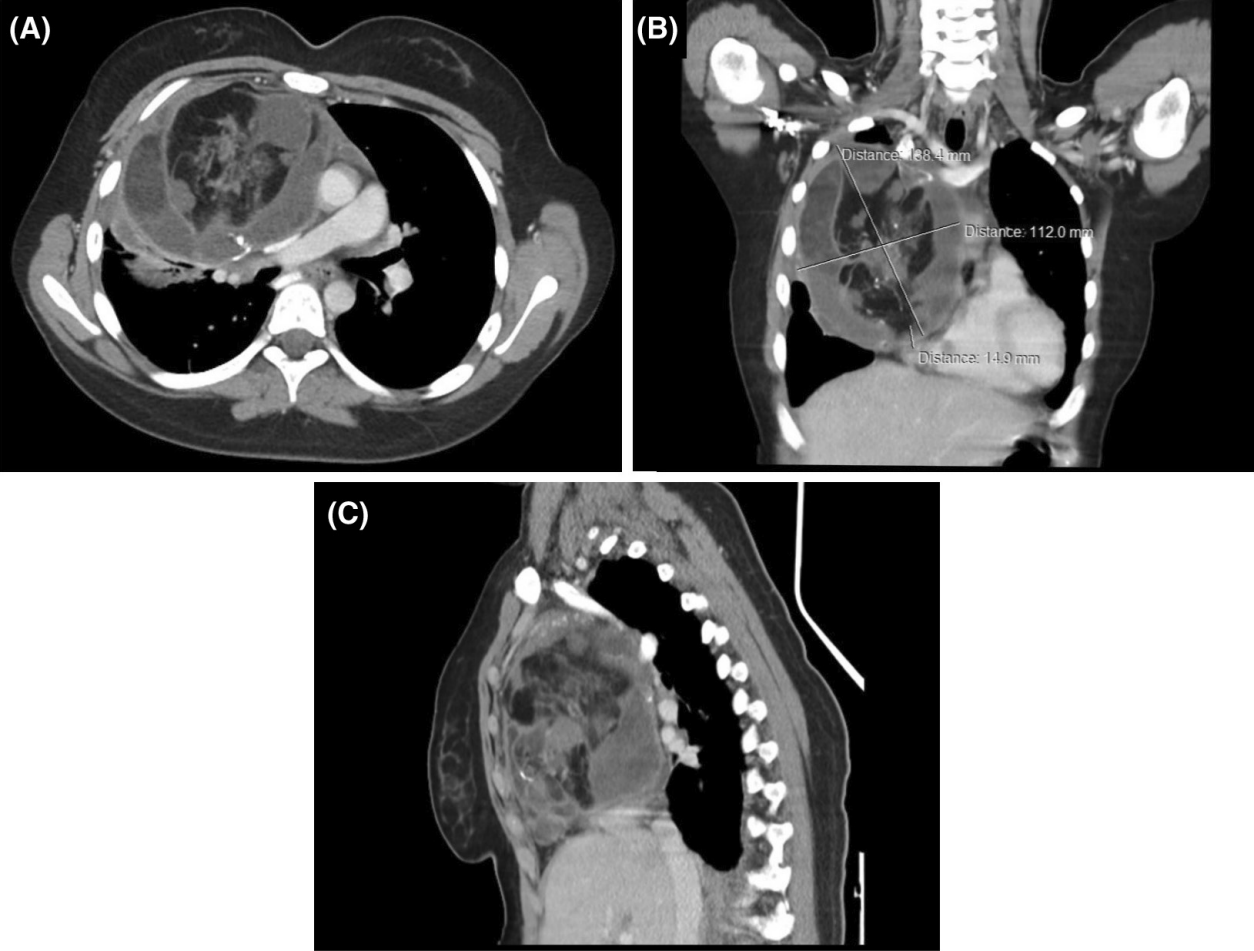
(A) Axial, (B) coronal, (C) sagittal CT section showing a well‐defined heterogeneous mass occupying the right part of the anterior mediastinum and a part of the right hemithorax, consisting of variable densities of fat, liquid, and bonny tissues.

Therefore, computed tomography (CT) was ordered and showed a (138.4 × 112 × 96) mm well‐defined heterogeneous mass occupying the right part of the anterior mediastinum and a part of the right hemithorax. The mass consisted of variable densities of fat, liquid, and bonny tissues that were separated by some septate (Figure 1). The mass caused compression on the adjacent mediastinal structures and the middle lobe of the right lung, which directed the diagnosis toward mature teratoma.

**FIGURE 2:**
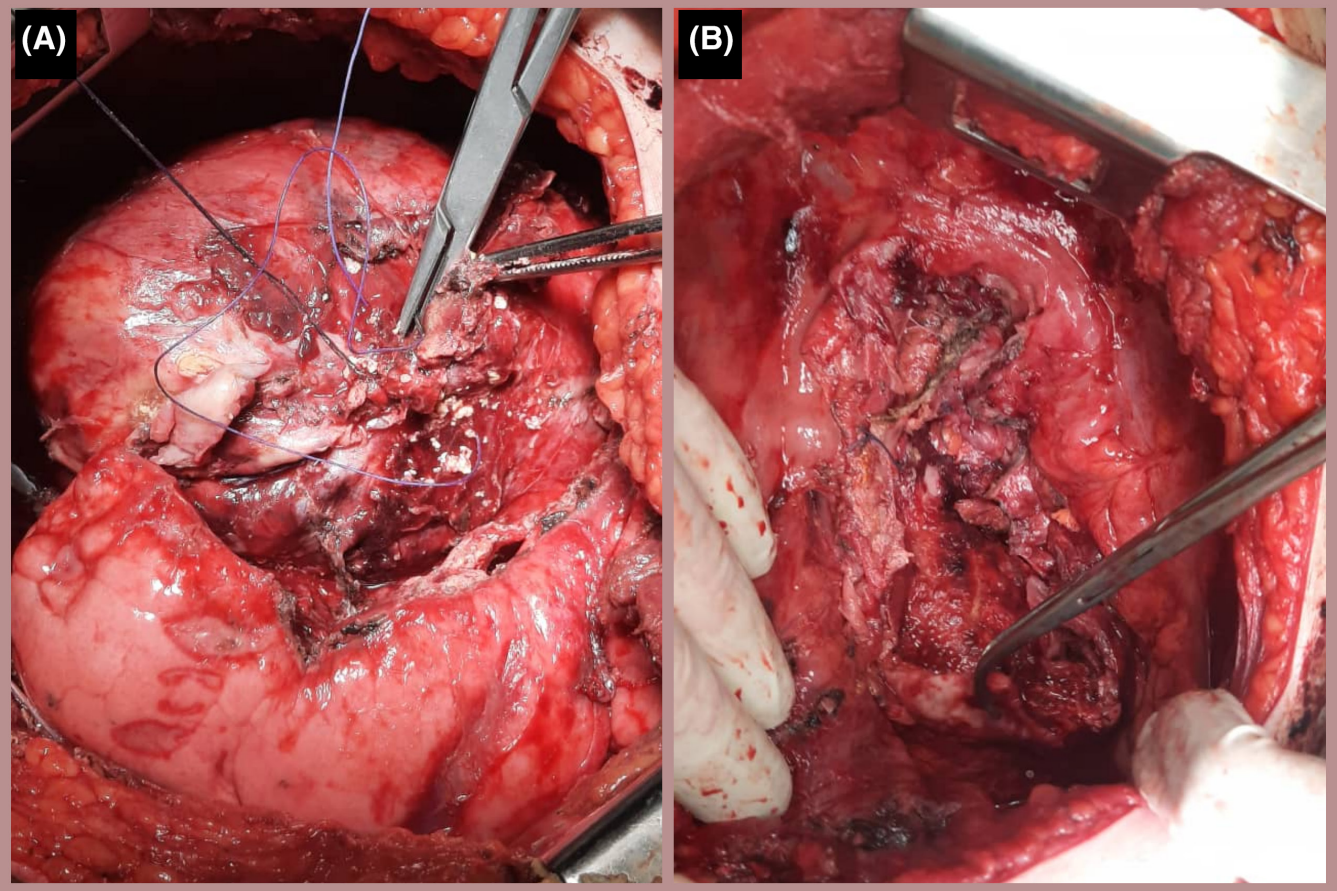
Intraoperative photograph showing: (A) The mass before resection and its compression on the right lung tissue and (B) the extremely adherent tissues to the pericardium which was retained after tumor resection to avoid cardiac injury.

After discussion, surgical treatment was recommended and a right hemithorax posterolateral incision was performed. There were no respiratory or circulatory problems during general anesthesia and intraoperatively. During the operation pleural, pericardium, and diaphragm adhesions were observed. The separation of the tumor from the normal tissues of the pleura and diaphragm was done but it was difficult to separate the tumor from the pericardium. The tumor was completely resected except for some tissues that were extremely adherent to the pericardium (Figure 2).

**FIGURE 3:**
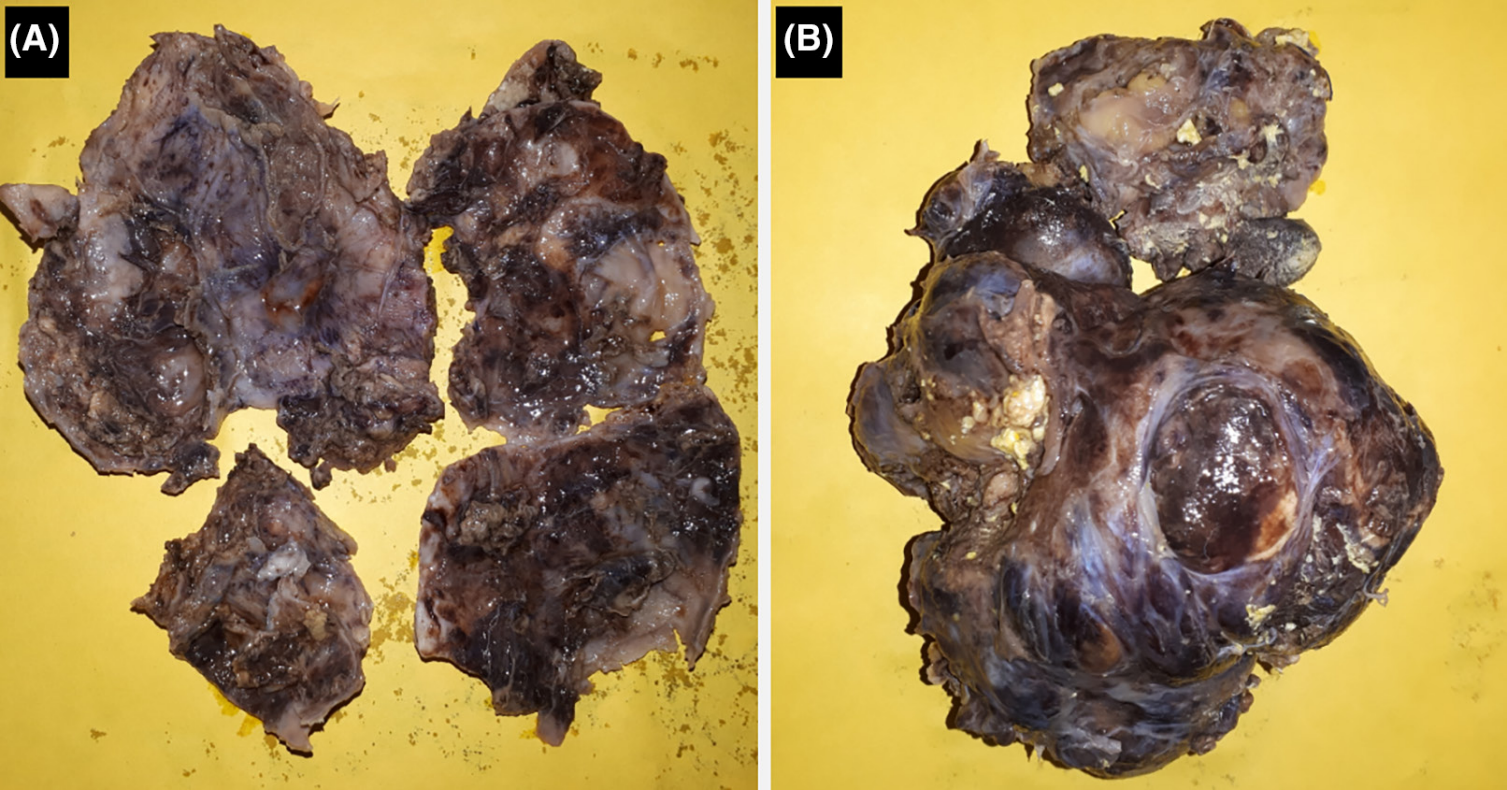
Gross inspection showing the wall (A) and contents (B) of the tumorous mass.

**FIGURE 4:**
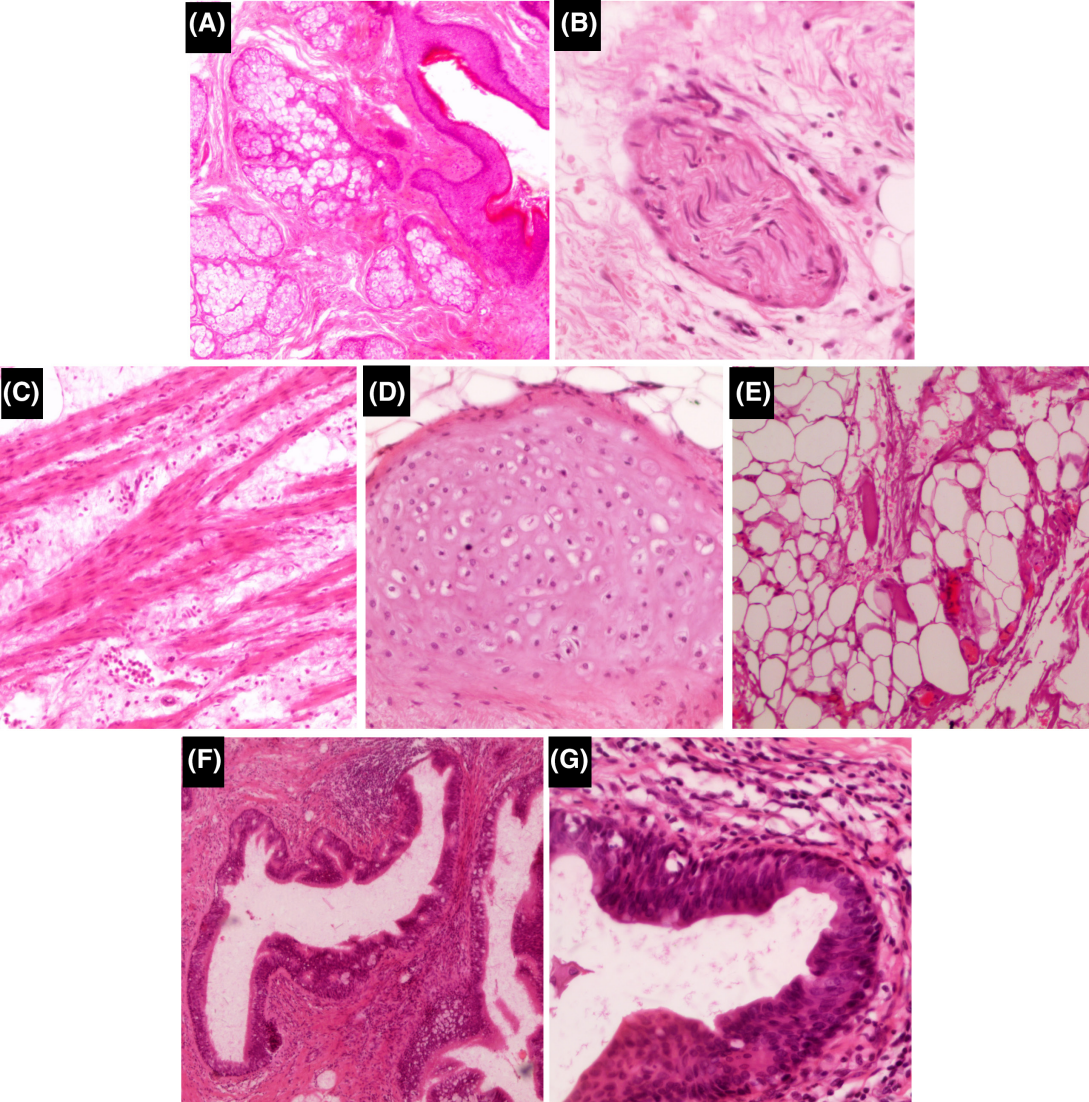
Microscopic findings with hematoxylin and eosin stain show mature tissues of the three germ cell layers: Ectoderm [Skin and sebaceous glands (×40) (A), nerve(×200) (B)]—Mesoderm [smooth muscle(×100) (C), chondral(×100) (D) adipose(×100) (E)]—Endoderm [glandular(×100) (F), esophageal(×200) (G)].

Gross inspection of the specimens revealed a fragmented mass, the largest fragment measures (10 × 9) cm **(**Figure 3 **)**. Microscopically, the mass consisted of mature tissues of ectoderm, mesoderm, and endoderm germ cell layers which confirmed the diagnosis of MMT (Figure 4).

## 4 CONCLUSION AND RESULTS (OUTCOME AND FOLLOW‐UP)

**FIGURE 5:**
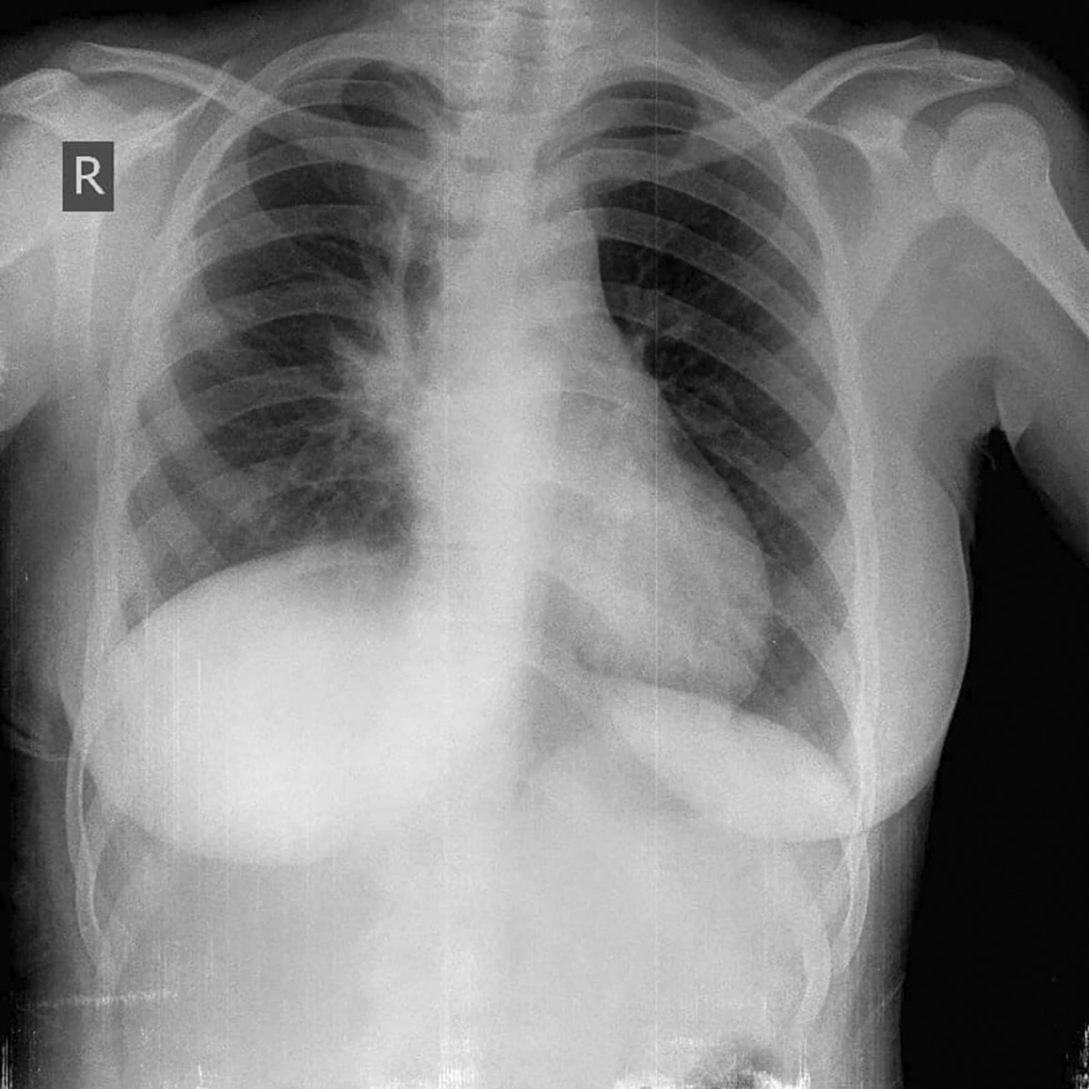
Chest x‐ray shows no evidence of recurrence.

The patient was discharged 3 days after the surgery. A follow‐up chest x‐ray was ordered 5 months after the surgery and showed no signs of recurrence. (Figure 5).

## 5 DISCUSSION

Teratomas are tumors that develop from embryonic cells which differentiate into tissues of all three germ cell layers. These tumors are typically diagnosed in the second and third decades of life. Teratomas can be classified as mature or immature based on the level of differentiation.^2^, ^3^, ^5^

MMT comprises approximately 3%–12% of mediastinal tumors in adults and 60%–70% of mediastinal germ cell tumors. MMT is typically detected in the anterior mediastinum which is the most common location of extragonadal germ cell tumors. In addition, the majority of teratoma tumors in the anterior mediastinum originally arise from the thymus or near thymus parenchyma, and intrapericardial or pericardial arising teratomas are uncommon.^1^, ^2^, ^3^

Clinically, MMT is often asymptomatic and can be discovered incidentally. Symptomatic cases usually present with chest pain, cough, dyspnea, dysphagia, or respiratory failure. The symptoms are usually caused by the growth of the tumor and its compression on the surrounding structures.^1^, ^5^

Chest X‐ray is the initial imaging procedure in diagnosing MMT, which generally appears as a rounded, heterogeneous, and well‐circumscribed mass.^1^, ^5^ In addition, chest CT is the main imaging tool for determining the exact location of the tumor and excluding infiltrative growth of mediastinal masses.^6^ The most common CT finding in MMT is the presence of a heterogeneous mass with soft tissue, fluid, fat, and calcium attenuation, seen in 39% of masses. Soft tissue is always visualized but rarely the dominant component in MMT whereas, fluid is the dominant component in 80% of MMTs.^7^ Compared to our case, a CT scan showed a well‐defined heterogeneous mass in the anterior mediastinum consisting of soft tissue, fluid, fat, and calcium.

As for the treatment, complete surgical resection is the standard treatment for MMT, it helps in establishing the diagnosis as well as ensuring a long‐term cure rate with a low risk of recurrence.^8^ The choice of surgical resection includes (thoracoscopic surgery with or without conversion to thoracotomy ‐ semi‐clamshell ‐ median thoracotomy ‐ simple neck collar incision ‐ posterolateral thoracotomy), which depends on the relationship between the surrounding tissue, tumor location, tumor size, invasion, and pleural adhesions.^9^ Specifically, posterolateral thoracotomy, which was performed in this reported case, can be selected for tumors located on one side of the mediastinum, large tumors, and invasion of the lung or pericardium.^10^

Histopathology findings in MMT show well‐differentiated tissues from all three germ cell layers. The most commonly observed components were skin or skin appendages and nerve tissue in the ectoderm; cartilage and fat in the mesoderm; and respiratory epithelium, pancreatic tissue, and gastrointestinal epithelium in the endoderm.^11^

Tumor resection could be incomplete when adhesions to the surrounding major organs exist, including major vessels in the mediastinum, heart, nerves, and lungs, and histopathology of the tumor shows that it is benign. So, regular follow‐up after the surgery is recommended to find any recurrence or malignant transformation of the tumor.^9^, ^11^

## 6 CONCLUSION

MMTs are germ cell tumors that develop into well‐differentiated tissues from all three germ cell layers. Adhesion to the surrounding major organs is uncommon and considered a surgical challenge which leads to incomplete tumor resection to avoid damage to the surrounding tissue. We report a rare case of MMT with pericardial adhesions and highlight the steps of diagnostic and surgical approaches.

## AUTHOR CONTRIBUTIONS

**Somar Mansour:** Writing – original draft; writing – review and editing. **Ali Badr:** Writing – original draft. **Majd Mansour:** Writing – original draft. **Mouhamad Badr:** Writing – original draft. **Ali Afif:** Writing – original draft. **Samer Rajab:** Writing – review and editing. **Zuheir Alshehabi:** Supervision; writing – review and editing.

## FUNDING INFORMATION

None.

## CONFLICT OF INTEREST STATEMENT

The authors declare that they have no competing interests.

## CONSENT

Written informed consent was obtained from the patient's parents to publish this report in accordance with the journal's patient consent policy.

## Data Availability

Data and material are available on reasonable request from the corresponding author.
